# Dietary effects on the development and population dynamics of the *Thelazia callipaeda* vector *Phortica okadai* revealed by age-stage, two-sex life table analysis

**DOI:** 10.1186/s13071-026-07408-y

**Published:** 2026-05-08

**Authors:** Zhenfu Chen, Rong Yan, Lingjun Wang, Juan Zhou, Bo Luo, Donghua Long, Rengze Yue, Hui Liu, Yujuan Shen

**Affiliations:** 1 NHC Key Laboratory of Parasite and Vector Biology, National Institute of Parasitic Diseases, Chinese Center for Diseases Control and Prevention (Chinese Center for Tropical Diseases Research), Shanghai, 200025 China; 2 Department of Parasitology, Zunyi Medical University, Zunyi, 563000 China

**Keywords:** *Phortica okadai*, *Thelazia callipaeda*, Age-stage two-sex life table, Laboratory rearing, Vector ecology

## Abstract

**Background:**

*Phortica okadai* (Diptera: Steganinae) is the primary vector of the zoonotic eyeworm *Thelazia callipaeda* (Nematoda: Spirurida). However, standardized laboratory rearing protocols for this vector are still lacking, which limits research on its biology and vector competence.

**Methods:**

We evaluated the effects of five diets (fermented apple, pear, banana, and two artificial diets) on life history traits of *P. okadai* using age stage, two sex life table analysis under controlled conditions (28 ± 1 °C, 75 ± 5% RH, 14:10 h L:D). Life table parameters and population dynamics were analyzed with TWOSEX-MSChart and TIMING-MSChart (100,000 bootstrap replicates).

**Results:**

All tested diets supported complete development. Fermented pear yielded the shortest pre adult duration (17.34 days), the highest fecundity (116.6 eggs per female), and the greatest intrinsic rate of increase (*r* = 0.0902/day). Population projection showed that 10 initial eggs on fermented pear produced more than 1,200 adults within 90 days, which was approximately 10 fold higher than on other diets.

**Conclusions:**

Fermented pear is the most suitable substrate for establishing laboratory colonies of *P. okadai*. These findings facilitate vector competence studies and indicate that pear orchards are potential high risk habitats for *T. callipaeda* transmission, supporting targeted One Health surveillance and control.

**Graphical Abstract:**

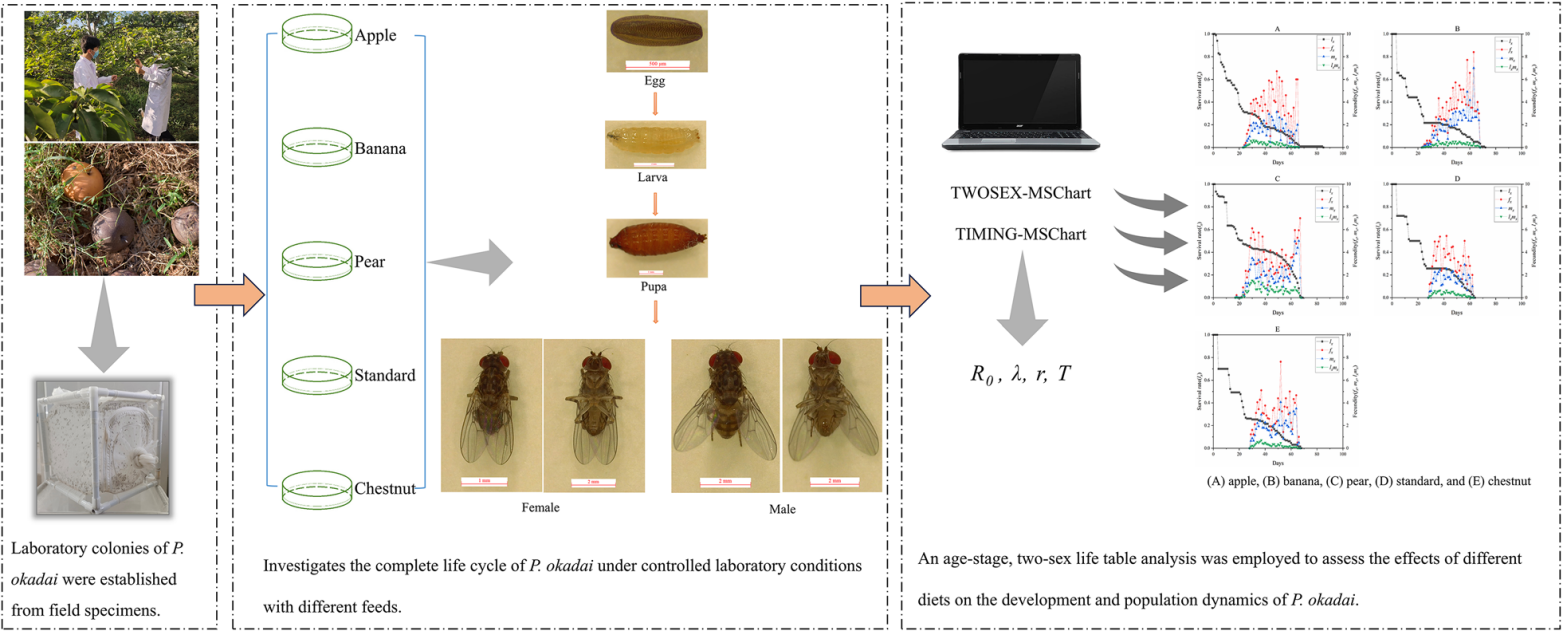

**Supplementary Information:**

The online version contains supplementary material available at 10.1186/s13071-026-07408-y.

*Phortica okadai* [1] (Diptera: Steganinae) (Máca, 1977) is a dual-threat species: an agricultural pest infesting fruit crops and the primary vector of the zoonotic nematode *Thelazia callipaeda* [2] (Nematoda: Spirurida) (Railliet and Henry, 1910), which causes ocular pathology in humans and animals [2–5]. Despite its significance in agriculture and public health, standardized laboratory rearing protocols for *P. okadai* are lacking, limiting research on its biology and vector competence [6, 7]. The age-stage, two-sex life table is a robust tool for quantifying demographic responses to environmental factors, making it ideal for evaluating dietary effects to optimize rearing [8–12]. This study aimed to identify the optimal diet for *P. okadai* and provide a validated rearing protocol, while identifying potential risk habitats for *T. callipaeda* transmission.

Adult *P. okadai* were field-collected from pear orchards in Zunyi, China (Additional file 1: Fig. S1) and domesticated for > 140 generations under controlled conditions (28 ± 1 °C, 75 ± 5% RH, 14:10 h L:D). Five diets were tested: three naturally fermented fruit substrates (apple, pear, and banana; cut into 1-cm^3^ cubes and subjected to ferment naturally at 28 ± 1 °C for 3 days in transparent plastic containers, 60 mm × 80 mm) and two artificial solid media were prepared on a weight/weight (w/w) percentage basis following Bernardini et al. [13]. The artificial media formulations were: Standard (84.3% water, 6.6% yeast, 4.4% sucrose, 0.7% agar, 3.3% cornmeal, 0.7% propionic acid) and Chestnut (84.3% water, 6.6% yeast, 4.4% sucrose, 0.7% agar, 2.6% chestnut flour, 0.7% banana (fresh fruit puree), 0.7% propionic acid). Thirty pairs of newly emerged adults were placed in each cage (20 × 20 × 20 cm^3^) for 12-h oviposition. For each dietary treatment, at least 100 eggs were collected from a single oviposition event. Eggs were transferred individually to plastic boxes (80 × 80 mm^2^) lined with damp cotton, and developmental progress (egg, larva, pupa, adult) and survival were monitored daily. Bootstrap resampling was applied to estimate standard errors for life table parameters. Upon emergence, adults were sexed and paired (1:1) to record fecundity until death. If female numbers exceeded males within a replicate, additional males of equivalent age from the same cohort were selected to ensure complete pairing. Age-stage, two-sex life tables were constructed using TWOSEX-MSChart [14–16], with key parameters including net reproductive rate (*R*_0_), intrinsic rate of increase (*r*), finite rate of increase (*λ*), and mean generation time (*T*). Population projections over 90 days were generated using TIMING-MSChart [17], and statistical differences were assessed via paired bootstrap tests (*P* < 0.05).

All five substrates supported complete development of *P. okadai*, but key life history traits and population parameters varied among diets (Additional file 1: Table S1). Egg duration (1.42–1.58 days) did not differ among diets (paired bootstrap test, *P* > 0.05). Larval development was shortest on pear (5.62 days) followed by apple (6.63 days), both being shorter than banana (7.70 days), standard (7.97 days), and chestnut (7.58 days) (paired bootstrap test, *P* < 0.05). Pre-adult duration was shortest on pear (17.34 days), while apple showed an intermediate duration (18.22 days), longer than pear but shorter than the other three diets (paired bootstrap test, *P* < 0.05). Adult longevity was longest on pear (36.37 days) and banana (36.08 days), exceeding apple (30.41 days) and chestnut (30.76 days) (paired bootstrap test, *P* < 0.05). Fecundity was highest on pear (116.64), exceeding apple (77.27), standard (93.19), and chestnut (72.44) diets (paired bootstrap test, *P* < 0.05). The adult pre-oviposition period (APOP) was shortest on pear (9.79 days) (paired bootstrap test, *P* < 0.05).

Population parameters indicated pear as the best-performing substrate in this study, with the highest net reproductive rate (*R*_0_ = 32.46 offspring/female), intrinsic rate of increase (*r* = 0.0902/day), and finite rate of increase (*λ* = 1.0944/day) and shortest mean generation time (*T* = 38.60 days) (Additional file 1: Table S2). Confidence intervals for *R*_0_, *r*, or* λ* overlapped among apple, banana, standard, and chestnut diets. Age-specific survival (*l*_*x*_) declined most gradually on pear, retaining the highest values until day 20, after which all diets showed a synchronized steep increase in adult mortality (Fig. 1). Reproduction (*m*_*x*_) began earliest on pear (day 17), followed by apple and banana (day 23), and standard and chestnut (day 28); the *f*_*x*_ curve nevertheless reached its absolute maximum on banana. The product *l*_*x*_*m*_*x*_ peaked on day 30 for pear, day 31 for apple, and day 37 for banana, standard and chestnut, indicating that the higher early fertility on pear compensated for its lower daily fecundity, whereas the other diets concentrated reproductive output later. Population projections showed adults emerged earliest on pear (day 15), with the population reaching 1230 adults by day 90—tenfold higher than apple (119 adults) and 40-fold higher than banana (30 adults) (Fig. 2). Detailed survival data, age-stage-specific life expectancy, and reproductive value are provided in Additional file 2: Figs. S2, S3, and S4.

**Fig. 1:**
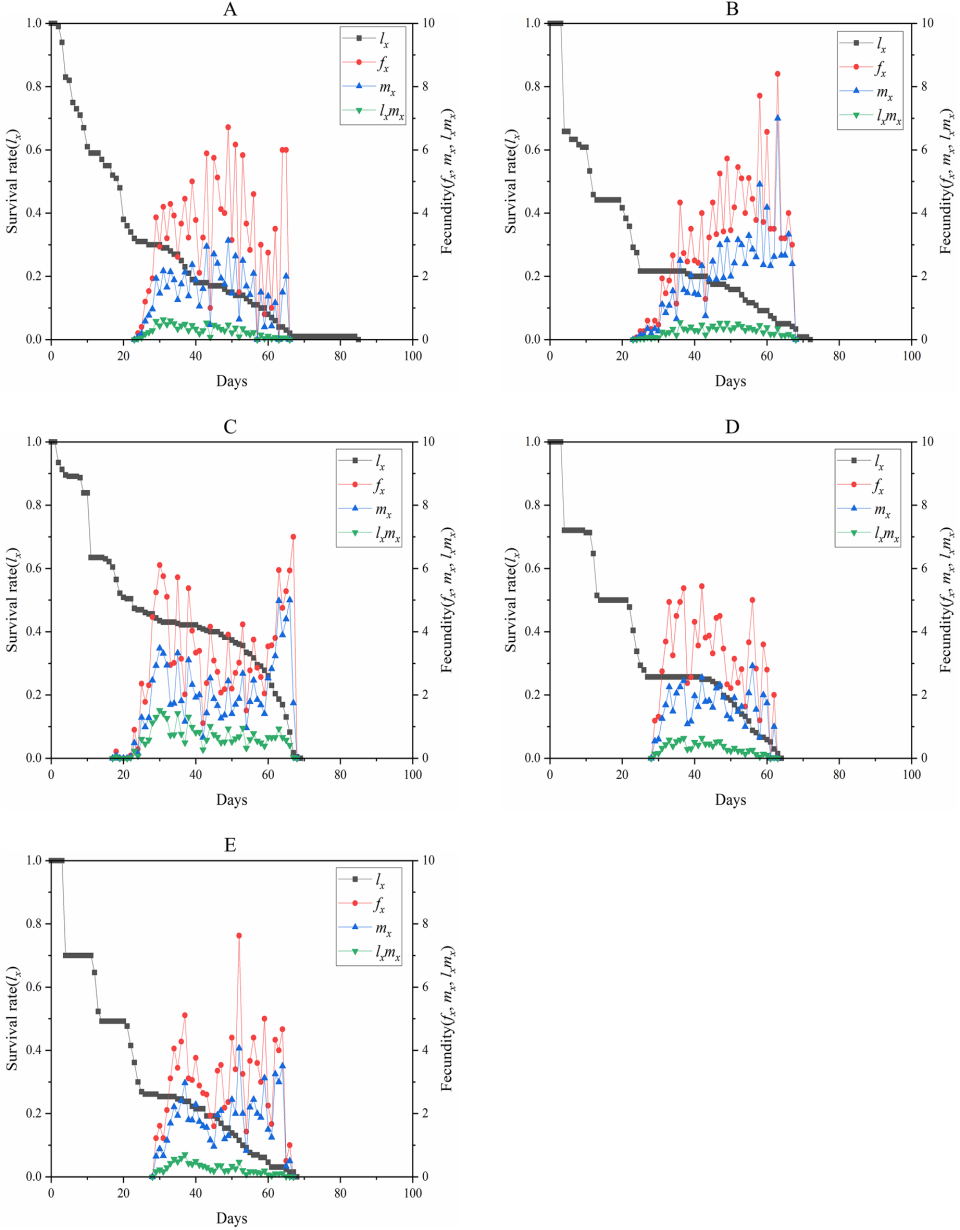
Age-specific survival rate (*l*_*x*_), age-stage fecundity of females (*f*_*x*_), age-specific fecundity of the cohort (*m*_*x*_) and age-specific maternity (*l*_*x*_*m*_*x*_) of *Phortica okadai* on different diets: **A** apple, **B** banana, **C** pear, **D** standard, and **E** chestnut

**Fig. 2:**
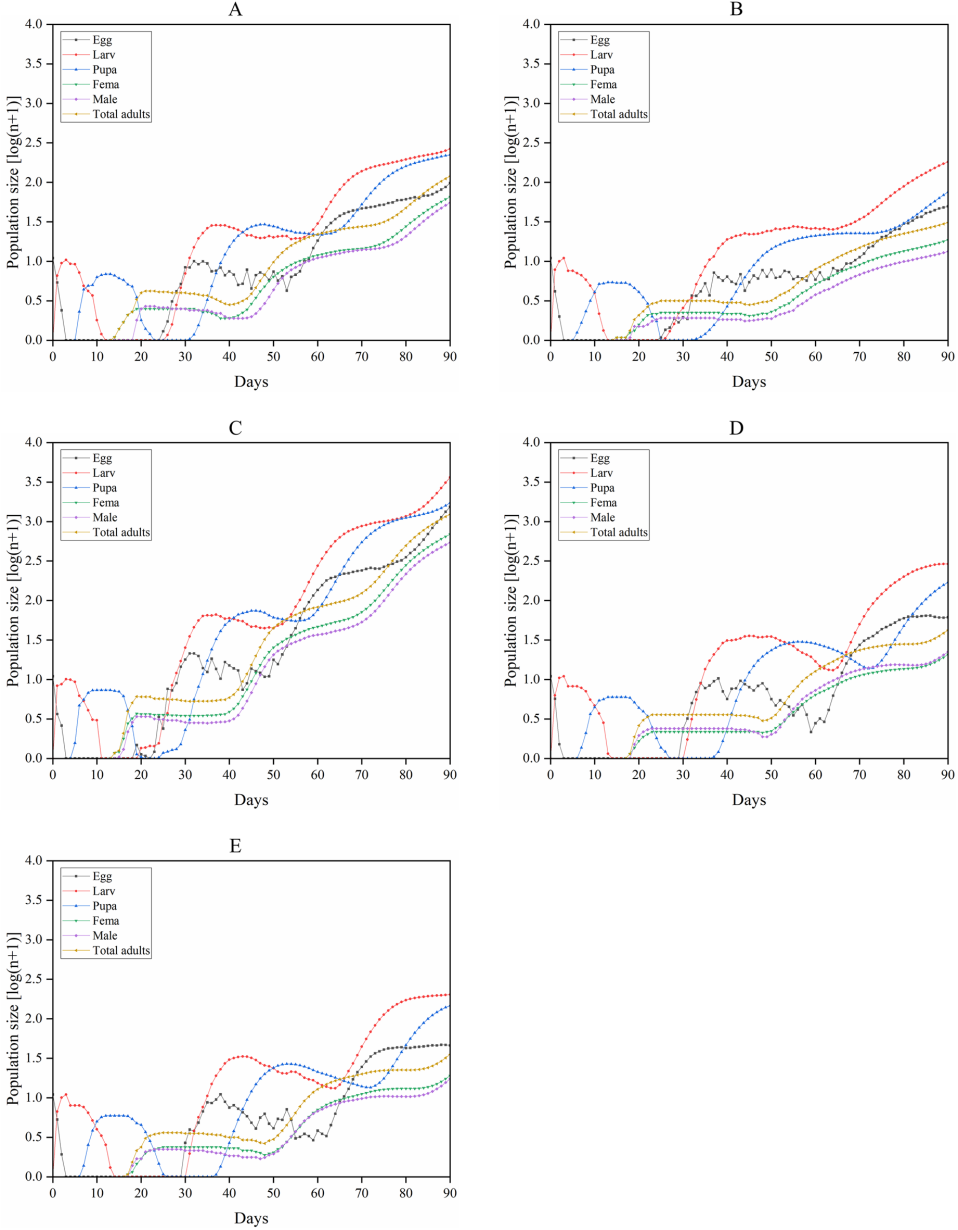
Population projection of *Phortica okadai* on different diets: **A** apple, **B** banana, **C** pear, **D** standard, and **E** chestnut. Projections initiated with ten eggs

Fermented pear emerged as the best-performing substrate for *P. okadai* in this study, as it accelerates development, improves fecundity, and boosts population growth potential. Its superiority is likely attributed to favorable nutritional composition (high fructose and sucrose), softened texture facilitating oviposition and larval foraging, and beneficial microbial communities that enhance nutrient assimilation [18–22]. Although fermented apple exhibited a comparable mean generation time (38.83 days), pear’s higher intrinsic rate of increase and fecundity make it more suitable for mass rearing of *P. okadai* in laboratory settings.

This optimized rearing substrate provides a standardized tool for establishing stable *P. okadai* colonies, laying a reliable foundation for subsequent studies on vector competence and the development of targeted control strategies. Pear and apple orchards, as natural habitats of *P. okadai*, constitute potential risk foci for *T. callipaeda* transmission and therefore require targeted surveillance [23]. Integrating fruit volatile-baited traps with host-associated cues could further improve the monitoring efficiency of *P. okadai* populations [24, 25]. A comprehensive One Health approach, integrating orchard surveillance, veterinary anthelmintic treatment of reservoir hosts (e.g., dogs and cats), and peri-urban vector suppression, is critical to mitigating the transmission risk of this zoonotic parasite [26–29].

However, a potential limitation of this study should be noted. The laboratory colony of *P. okadai* used in this study had been maintained exclusively on fermented pear for more than 140 generations before the experiments, which may have led to laboratory adaptation. Long-term rearing on a single diet could induce physiological, behavioral, and gut microbiome-associated changes that improve performance on the original maintenance medium while imposing fitness costs on alternative diets [30–32]. Therefore, the superior demographic performance on fermented pear may partly reflect laboratory adaptation to long-term rearing, rather than only the intrinsic nutritional advantage of this diet. To reduce such effects and obtain more robust conclusions, future studies could use colonies that have been acclimated to each test diet for multiple generations before formal experiments.

## Supplementary Information

Additional file 1: Table S1. Means ± standard errors of pre-adult duration, adult longevity, APOP, TPOP, oviposition days, oviposition period, and fecundity (Nf) of *Phortica okadai* on different diets. Table S2. Means ± standard errors of net reproductive rate (*R*_0_), intrinsic rate of increase (*r*), finite rate of increase (*λ*), and mean generation time (*T*) of *Phortica okadai* on different diets.

Additional file 2: Fig. S1. Map of the sampling sites of *Phortica okadai* in Zunyi, China. The red regions indicate the sampling area. Fig. S2. Age-stage-specific survival rate (*S*_*xj*_) of Phortica okadai on different diets: (A) apple, (B) banana, (C) pear, (D) standard, and (E) chestnut. *S*_*xj*_ represents the probability that a newly laid egg survives to age *x* and stage *j*. Fig. S3. Age-stage-specific life expectancy (*e*_*xj*_; the survival probability of an individual of age *x* and stage *j*) of *Phortica okadai* on different diets: (A) apple, (B) banana, (C) pear, (D) standard, and (E) chestnut. Fig. S4. Reproductive value (*v*_*xj*_; the contribution of an individual of age *x* and stage *j* to future population growth) of *Phortica okadai* on different diets: (A) apple, (B) banana, (C) pear, (D) standard, and (E) chestnut.

## Data Availability

All supporting data and protocols have been provided within the article or through Supplementary information.

## Abbreviations

APOP: Adult pre-oviposition period
TPOP: Total pre-oviposition period
JH: Juvenile hormone
IPM: Integrated pest management
L:D: Light:dark cycle
