# Supplementary material for: Dietary effects on the development and population dynamics of the Thelazia callipaeda vector Phortica okadai revealed by age-stage, two-sex life table analysis
Source: Parasit Vectors. 2026 May 8;19:268. doi: 10.1186/s13071-026-07408-y (PMC13321572; doi:10.1186/s13071-026-07408-y)
Supplement: Supplementary file 1 — Additional file 1: Table S1. Means ± standard errors of pre-adult duration, adult longevity, APOP, TPOP, oviposition days, oviposition period, and fecundity (Nf) of Phortica okadai on different diets. Table S2. Means ± standard errors of net reproductive rate (R0), intrinsic rate of increase (r), finite rate of increase (λ), and mean generation time (T) of Phortica okadai on different diets. [file 13071_2026_7408_MOESM1_ESM.docx]

**Table S1**. Means ± standard errors of pre-adult duration, adult longevity, APOP, TPOP, oviposition days, oviposition period, and fecundity (*N*f) of *Phortica okadai* on different diets.

| Developmental Stage | Developmental Period at Different Food | | | | | | | | | |
| --- | --- | --- | --- | --- | --- | --- | --- | --- | --- | --- |
|  | Apple | | Pear | | Banana | | Standard | | Chestnut | |
|  | N | Mean±SE | N | Mean±SE | N | Mean±SE | N | Mean±SE | N | Mean±SE |
| Egg duration (d) | 100 | 1.58±0.07a | 230 | 1.43±0.05a | 120 | 1.42±0.06a | 136 | 1.52±0.05a | 130 | 1.52±0.06a |
| Larva duration (d) | 59 | 6.63±0.17b | 146 | 5.62±0.10c | 53 | 7.70±0.17a | 68 | 7.97±0.18a | 64 | 7.58±0.15a |
| Pupa duration (d) | 32 | 11.62±0.26a | 120 | 10.84±0.10b | 26 | 10.69±0.3b | 35 | 10.97±0.25a | 34 | 11.09±0.31a |
| Preadult duration (d) | 32 | 18.22±0.34b | 120 | 17.34±0.11c | 26 | 20.65±0.47a | 35 | 20.26±0.23a | 34 | 20.21±0.33a |
| Adult duration (d) | 32 | 30.41±2.54b | 120 | 36.37±1.40a | 26 | 36.08±1.87a | 35 | 34.17±0.99a | 34 | 30.76±1.65b |
| Female total longevity (d) | 15 | 48.47±3.10b | 64 | 58.06±1.74a | 15 | 57.73±2.60a | 16 | 56.56±1.20a | 18 | 52.94±1.99b |
| Male total longevity (d) | 17 | 48.76±3.98a | 56 | 48.73±2.04a | 11 | 55.36±2.83a | 19 | 52.63±1.49a | 16 | 48.75±2.66a |
| Adult total longevity (d) | 32 | 48.63±2.52b | 120 | 53.71±1.39a | 26 | 56.73±1.89a | 35 | 54.43±1.02a | 34 | 50.97±1.65b |
| Adult pre-oviposition period (APOP) (d) | 15 | 11.27±0.27a | 64 | 9.79±0.36b | 15 | 10.53±0.31a | 16 | 10.81±0.19a | 18 | 11.28±0.25a |
| Total pre-oviposition period (TPOP) (d) | 15 | 27.87±0.58b | 64 | 27.02±0.43b | 15 | 31.00±0.74a | 16 | 31.19±0.38a | 18 | 31.89±0.63a |
| Oviposition days (d) | 15 | 13.20±2.16b | 64 | 19.33±0.90a | 15 | 18.87±2.19a | 16 | 17.69±0.99a | 18 | 14.83±1.43b |
| Oviposition period (d) | 15 | 19.00±9.37a | 64 | 33.72±2.19a | 15 | 25.20±11.60a | 16 | 23.94±8.78a | 18 | 19.72±7.35a |
| Fecundity (*Nf*) (eggs) | 15 | 77.27±14.35b | 64 | 116.64±8.63a | 15 | 95.73±12.56a | 16 | 93.19±5.50b | 18 | 72.44±8.21c |

Note：SEs were estimated by using the bootstrap technique with 100,000 resampling. The means followed by a different letter in the same row are significantly different between cultivars determined by using the paired bootstrap test based on the confidence interval of difference (*P* < 0.05).

**Table S2.** Means ± standard errors of net reproductive rate (*R₀*), intrinsic rate of increase (*r*), finite rate of increase (*λ*), and mean generation time (*T*) of *Phortica okadai* on different diets.

| Population Parameters | Different Feeds | | | | |
| --- | --- | --- | --- | --- | --- |
|  | Apple | Pear | Banana | Standard | Chestnut |
| Net reproductive rate (*R_0_*) | 11.59±3.47b | 32.46±4.19a | 11.97±3.27b | 10.96±2.65b | 10.03±2.46b |
| Intrinsic rate of increase (*r*) (d^-1^) | 0.0629±0.0081b | 0.0902±0.0039a | 0.0540±0.0065b | 0.0575±0.0063b | 0.0551±0.0061b |
| Finite rate of increase (*λ*) (d^-1^) | 1.0650±0.0086b | 1.0944±0.0043a | 1.0555±0.0068b | 1.0592±0.0066b | 1.0566±0.0064b |
| Mean generation time (*T*) (d^-1^) | 38.83±1.20c | 38.60±0.59c | 45.92±1.27a | 41.66±0.62b | 41.86±1.05b |

Note: SEs were estimated by using the bootstrap technique with 100,000 resampling. The means followed by different letters in the same row were significantly different according to the paired bootstrap test based on the confidence interval of differences at the 5% significance level.
