# Supplementary material for: Dietary effects on the development and population dynamics of the Thelazia callipaeda vector Phortica okadai revealed by age-stage, two-sex life table analysis
Source: Parasit Vectors. 2026 May 8;19:268. doi: 10.1186/s13071-026-07408-y (PMC13321572; doi:10.1186/s13071-026-07408-y)

**Fig. S1** Map of the sampling sites of *Phortica okadai* in Zunyi, China. The red regions indicate the sampling area.

**
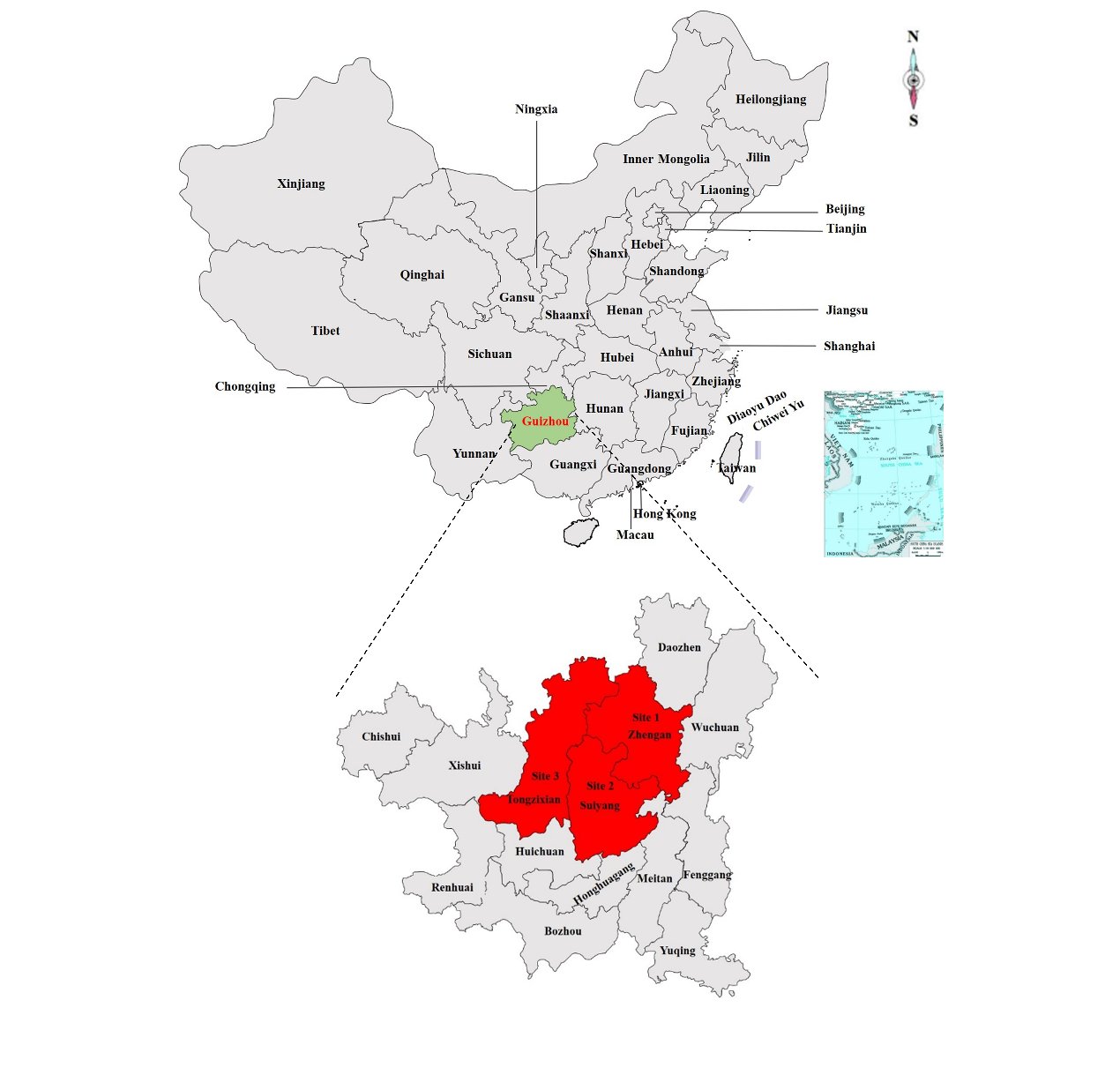
**

**Fig. S2** Age-stage-specific survival rate (*S_xj_*) of *Phortica okadai* on different diets: (A) apple, (B) banana, (C) pear, (D) standard, and (E) chestnut. *Sₓⱼ* represents the probability that a newly laid egg survives to age *x* and stage *j*.


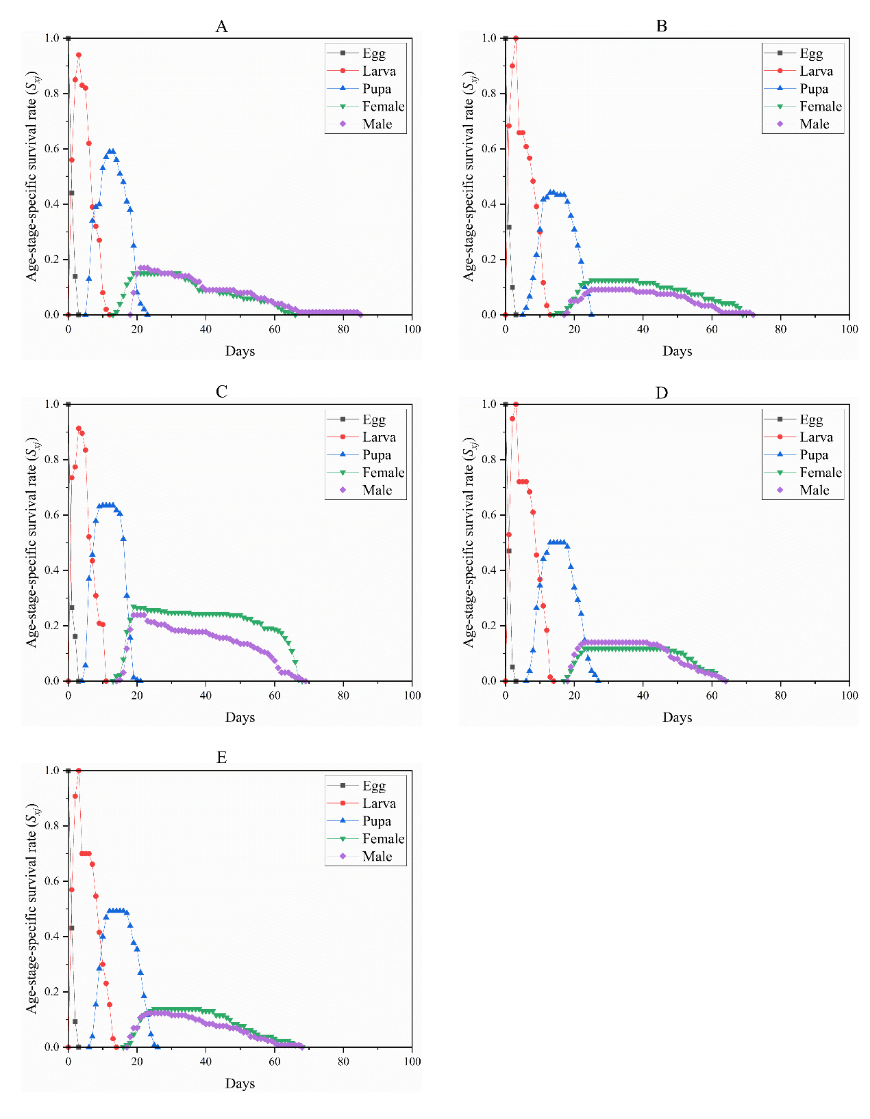


**Fig. S3** Age-stage-specific life expectancy (*e_xj_*; the survival probability of an individual of age *x* and stage *j*) of *Phortica okadai* on different diets: (A) apple, (B) banana, (C) pear, (D) standard, and (E) chestnut.


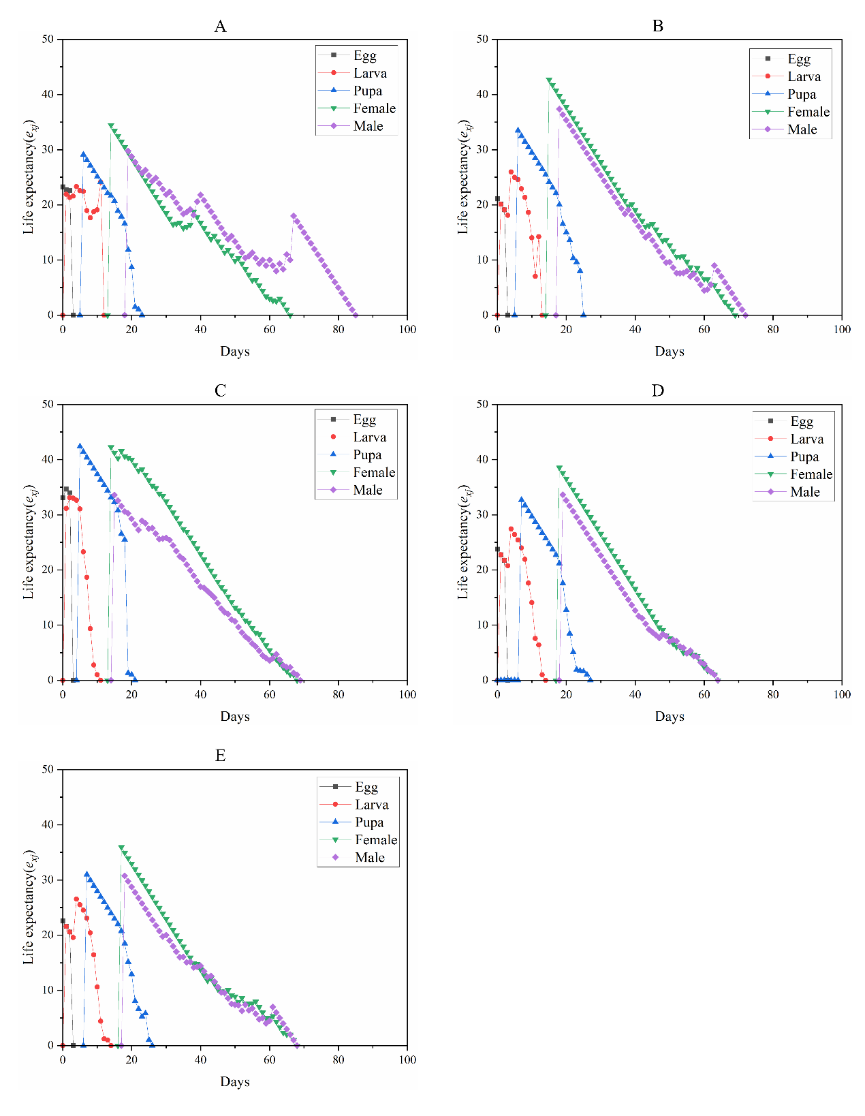


**Fig. S4** Reproductive value (*v_xj_*; the contribution of an individual of age *x* and stage *j* to future population growth) of *Phortica okadai* on different diets: (A) apple, (B) banana, (C) pear, (D) standard, and (E) chestnut.


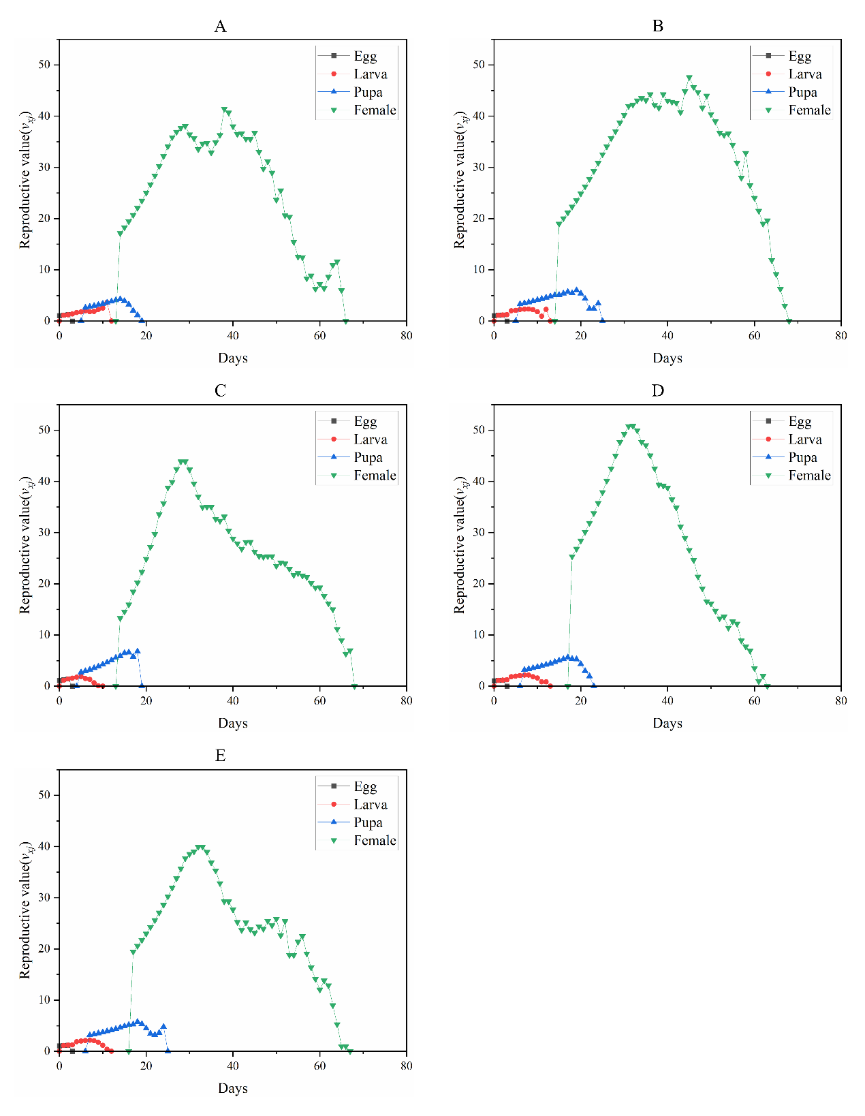

Supplement: Supplementary file 2 — Additional file 2: Fig. S1. Map of the sampling sites of Phortica okadai in Zunyi, China. The red regions indicate the sampling area. Fig. S2. Age-stage-specific survival rate (Sxj) of Phortica okadai on different diets: (A) apple, (B) banana, (C) pear, (D) standard, and (E) chestnut. Sxj represents the probability that a newly laid egg survives to age x and stage j. Fig. S3. Age-stage-specific life expectancy (exj; the survival probability of an individual of age x and stage j) of Phortica okadai on different diets: (A) apple, (B) banana, (C) pear, (D) standard, and (E) chestnut. Fig. S4. Reproductive value (vxj; the contribution of an individual of age x and stage j to future population growth) of Phortica okadai on different diets: (A) apple, (B) banana, (C) pear, (D) standard, and (E) chestnut. [file 13071_2026_7408_MOESM2_ESM.docx]
